# Transcatheter Tricuspid Valve Interventions: A Triumph for Transcatheter Procedures?

**DOI:** 10.3390/life13061417

**Published:** 2023-06-20

**Authors:** Kyriakos Dimitriadis, Nikolaos Pyrpyris, Konstantinos Aznaouridis, Panagiotis Iliakis, Aggeliki Valatsou, Panagiotis Tsioufis, Eirini Beneki, Emmanouil Mantzouranis, Konstantina Aggeli, Eleftherios Tsiamis, Konstantinos Tsioufis

**Affiliations:** First Department of Cardiology, School of Medicine, National and Kapodistrian University of Athens, Hippokration General Hospital, 115 27 Athens, Greece; npyrpyris@gmail.com (N.P.);; conazna@yahoo.com (K.A.); panayiotisiliakis@gmail.com (P.I.); ang.valatsou@gmail.com (A.V.); panos.tsioufis@gmail.com (P.T.); e.beneki@hotmail.com (E.B.); mantzoup@gmail.com (E.M.); dina.aggeli@gmail.com (K.A.); ltsiamis@otenet.gr (E.T.); ktsioufis@gmail.com (K.T.)

**Keywords:** tricuspid regurgitation, transcatheter edge-to-edge repair, tricuspid valve replacement, interventional cardiology

## Abstract

Tricuspid regurgitation (TR) is a common valvular pathology, estimated to affect 1.6 million people in the United States alone. Even though guidelines recommend either medical therapy or surgical treatment for TR, the misconception of TR as a benign disease along with the high mortality rates of surgical intervention led to undertreating this disease and commonly describing it as a “forgotten” valve. Recently, the development of transcatheter interventions for TR show promising potential for use in the clinical setting. There are currently few approved and numerous tested percutaneously delivered devices, which can be categorized, based on their mechanism of action, to either valve repair or valve replacement procedures. Both procedures were tested in clinical trials and show an echocardiographic reduction in TR sustained for at least 1 year after the procedure, as well as symptom relief and functional improvement of the patients. Device selection should be personalized, taking into consideration the anatomy of each valve and the available options at each heart center. Moreover, appropriate patient selection and timing of the procedure are also crucial for the success of the procedure. In this review, we analyze the clinical trials available for all devices currently approved or tested, aiming to provide a comprehensive summary of the most recent evidence in the field of transcatheter TR interventions.

## 1. Introduction

Tricuspid regurgitation (TR) is one of the most common valvular pathologies, being estimated as second of all valvular pathologies in frequency in the Framingham study [1]. The following studies found the prevalence of greater than moderate TR to be 0.55% in their study population and strongly associated with female gender and increased age [2], while the OxValve study found that 2.7% of their study population had significant or severe TR [3]. In the US, the prevalence of TR was estimated to be 1.6 million, with annual new TR diagnosis being 250,000; however, lower than 8000 TR surgeries were completed [4,5]. More recently, the UK Biobank study reported an incidence rate of 2.0 per 10,000 person–years and a mortality rate of 0.51 deaths per 10,000 person–years [6]. Regarding economic burden, a recent study showed that TR, irrespective of heart failure presence, is associated with higher rates of all-cause hospitalization, hospital days, and healthcare-associated expenditure [7].

It is well established that significant TR is associated with a higher disease burden, and worse cardiovascular outcomes. In a retrospective study [8] analyzing 5223 patients, greater TR severity was correlated with higher mortality rates. Furthermore, a subanalysis of the COAPT trial in patients with TR and mitral regurgitation (MR) showed that individuals with MR and moderate or severe TR had higher NYHA class and BNP levels, as well as more severe MR, highlighting the overall worse clinical outcomes in patients with TR and MR coexistence [9]. Finally, in the setting of heart failure with reduced ejection fraction, TR was associated with a more severe heart failure presentation as well as worse survival independently of other baseline markers [10]. 

Even though TR interventions remained low in past years, studies showed an increasing number of TR surgery recently, indicating the increased interest in the valve historically known as “the forgotten valve”. In the 2021 European Society of Cardiology (ESC) /European Association for Cardio-Thoracic Surgery (EACTS) guidelines for the management of valvular disease [11], the treatment indicated for TR is surgery and medical therapy for those unsuitable for surgical intervention. It is well established, however, that surgical treatment is associated with high periprocedural and in-hospital fatality, with large studies mentioning mortality rates ranging from 8.2% to 27.6% [12,13]. Described reasons for these outcomes are the advanced stages of disease at which the patients are referred for surgical consultation [14]. Therefore, despite being suggested by guidelines, surgery still remains underused [15]. 

Following recent advances in the transcatheter management of other valvular pathologies, increasing interest was shown in the interventional management of TR. Therefore, novel devices are currently being investigated in terms of efficacy and safety. These devices can be divided into two broad categories, regarding their mechanism of action, to either valve repair or valve replacement interventions. Clinical trials provided insightful information about the potential use of such devices in the everyday clinical setting. The aim of this review is to address the need for intervention in TR, as well as describe all available data on the use of transcatheter tricuspid valve interventions for the management of TR.

## 2. Pathophysiology of Tricuspid Regurgitation

TR can be pathophysiologically categorized as primary TR, where there is an intrinsic abnormality in the tricuspid valve, and secondary or functional TR, where the regurgitation is a result of the enlargement of the right heart chambers, resulting in a subsequent dilation of the tricuspid annulus and regurgitation [16].

Primary TR is a result of an either preexisting congenital or acquired defect in the tricuspid valve. The most commonly congenital pathology is Ebstein’s anomaly, a rare disease accounting for 0.3–0.5% of congenital heart defects [17], affecting 0.39–0.72 children per 10,000 births [18,19]. As an embryogenic anomaly, it is characterized by failure of delamination of the tricuspid valve, with the septal and posterior leaflets being displaced towards the apex of the right ventricle, resulting in displacement of the tricuspid functional annulus. The most common cause for primary TR is rheumatic heart disease [16]. Rheumatic TR is present in 7.7% of rheumatic heart disease patients, and in most cases, it is associated with a concomitant pathology of the mitral valve [20]. A restrictive and regurgitant phenotype is commonly encountered, as a pure stenotic or regurgitant pathology is rare [21]. Other causes of primary TR include carcinoid syndrome [22] and right-sided infectious endocarditis, which is more rarely encountered (5–10% of all endocarditis cases); however, 90% of its cases develop TR [23]. Finally, iatrogenic primary TR is a known complication of the implantation of either a pacemaker or defibrillator device, caused either by direct mechanical damage to the tricuspid valve leaflets or delayed right ventricular activation and alteration of the right ventricular morphology [24].

Secondary or functional TR is not commonly associated with the valve itself rather than the negative remodeling of the right ventricle, which results in dilation of the tricuspid annulus and misalignment of the valvular leaflets [25]. Conditions that could result in this disease are left heart valve pathologies and pulmonary hypertension, which by increasing backward pressure, promote ventricular and atrial remodeling as well as annular dilation. In specific, there is a close relation of TR with other valvular pathologies, as studies show that TR coexists with severe mitral regurgitation in 30–50% of patients and with severe aortic stenosis in 25% of patients [26]. Additionally, ischemic mitral regurgitation is strongly associated with development and progression of TR, while TR severity is proportional to the extent of the regurgitation [27]. Furthermore, studies showed that coexistence of TR at the time of surgery for mitral or aortic regurgitation adversely affects short- and long-term outcomes [28,29]. Increased risk is also evident in the coexistence of TR and low-flow, low-gradient aortic stenosis with reduced left ventricular ejection fraction [29]. Therefore, potential transcatheter management of coexisting valvular pathologies at the same time could be beneficial and improve patients’ outcomes; however, relevant data from clinical studies are still limited. Atrial fibrillation can also be accounted for TR by creating an annular dilation directly and not by negative remodeling of the right ventricle [30]. Secondary TR is the most common type of TR, with more than 90% of patients having functional TR, as shown by Mutlak et al. in an echocardiography-based study [31]. 

## 3. Need for Interventional Therapies–Transcatheter Intervention Options

**Figure 1:**
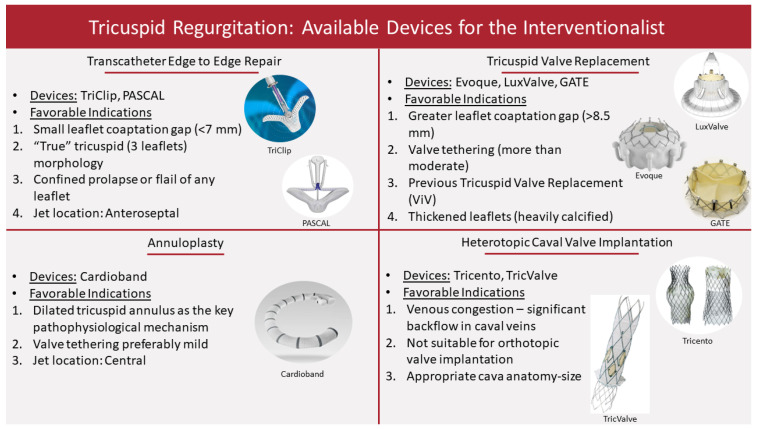
There are currently four techniques for the transcatheter management of tricuspid regurgitation, which subsequently have numerous currently tested or approved devices that the interventionalist can choose. Each technique, based on anatomical considerations, has some favorable indications of use, mostly depending on the technique-specific repair mechanisms.

It is well established thus far that TR is associated with increased disease burden and mortality rates [9,32]. Despite being commonly left untreated, TR is not considered anymore a benign disease and treatment should be offered appropriately. While surgical intervention remains underused, there is a need for an efficient and easily accessible treatment. Following the success of percutaneous interventions for the aortic and mitral valve, recent technological advances made transcatheter options for the management of TR available. There are currently several categories of devices tested or approved for use in TR (Figure 1), which will be described below.

### 3.1. Leaflet Approximation

Leaflet approximation is the most well-studied procedure thus far. The technique, also known as transcatheter tricuspid edge-to-edge repair (TEER), is aiming to approximate the leaflets of the tricuspid valve via implanting clips and coapting two of the leaflets. There are two TEER devices currently approved for use in TR: The TriClip, as derived by the device used for mitral regurgitation (MitraClip), and PASCAL. Both devices have established efficacy and safety profiles throughout clinical trials as depicted in detail in Table 1 and are approved for clinical use (CE mark).

#### 3.1.1. TriClip (Previously MitraClip)

Nickenig et al. [33] were the first to study use of a tricuspid leaflet approximation technique for TR using the MitraClip device. They included 64 patients with symptoms of heart failure (HF) and severe tricuspid regurgitation (TR) and implanted a MitraClip device with a 97% success rate. No intraprocedural deaths or major complication occurred. At 30 days after the procedure, TR was significantly reduced, with TR grade being reduced by at least 1 grade in 91% of the patients. NYHA class and a 6-minute walking test (6MWT) were also improved in this cohort, suggestive of the positive impact of the intervention in the functional status of the patients. Following studies from Orban et al. [34] and Braun et al. [35], both examining the use of MitraClip in small cohorts of patients with severe TR, also showed significant improvements in TR grade and functional status, as indicated by the improvement of NYHA class and 6MWT distanced.

Mehr et al. [36] studied 249 patients from the TriValve registry receiving a MitraClip for TR. The technical success rate was 96% while the acute procedural success, defined as a TR grade lower or equal to 2, was achieved in 77% of patients. The procedure decreased the proportion of patients with a TR grade greater than 3 from 97% pre-procedurally to 23% before discharge (*p* < 0.001), while TR reduction of at least 1 grade was reported in 89% of patients and improvement by at least 1 NYHA class was observed in 72% of patients. At 1 year, patients with a TR grade greater than 3 were significantly lower (baseline 97%; follow-up 28%; *p* < 0.001), while 69% of patients were NYHA class I/II. The estimated rate of combined mortality and rehospitalization for heart failure was 34.7%. 

Lurz et al. [37], in the TRILUMINATE trial, studied a total of 85 patients, receiving a TriClip for symptomatic TR. At follow-up, 87% of subjects had a sustained TR reduction of at least 1 grade after 1 year, with 70% of subjects having moderate or less TR, as compared to 8% at baseline and 60% at 30 days. Specifically, 56% (22 of 39) of subjects with baseline massive or torrential TR achieved moderate or less TR at 1 year, with 90% achieving at least a 1-grade reduction in TR. Subjects classified as NYHA functional class I/II increased from 31% at baseline to 83% at 1 year (*p* < 0.0001). The 6MWT distance was also significantly increased. Among all subjects at 1 year follow-up, the hospitalization rate decreased from 1.30 to 0.78 events/patient–year (*p* = 0.0030)

More recently, undergoing clinical trials presented their preliminary results in the clinical trial sessions of cardiology congresses. Specifically, Lurz et al. [38] presented at PCR London Valves 2021 the 30-day results of the bRIGHT study, the first real-world study, having enrolled 300 patients. In the preliminary results presented, the implantation success rate is 98%. At the 30 days follow-up, 71% of patients had moderate or less TR (*p* < 0.0001) and 78% were NYHA class I/II (*p* < 0.0001). In terms of safety, the procedure was found to be safe, as 1% of patients experienced a major adverse event during the 30 days follow-up. At PCR London Valves 2022, the 1-year results of bRIGHT were also presented [39]. At 1 year, 86% of patients had moderate or less TR, while the improvement in NYHA class and KCCQ was maintained throughout the year. A total of 11.5% of patients experienced a major adverse event. The mortality rate was 11.0%, while a 44% reduction in heart failure hospitalizations was noted. Moreover, D. Adams presented at TCT 2022 the results of 30 days of the device arm of TRILUMINATE-Pivotal trial [40]. In specific, 97 patients received a TriClip and were followed up for 30 days. The implantation success rate was 99%, while at 30 days, 74% had less than moderate TR and 67% had a reduction in TR class ≥ 2 grades. Moreover, 76% of patients were NYHA I/II at 30 days, in comparison to 32% at baseline (*p* < 0.0001). The procedure was safe, with a 1% mortality rate. A total of 7.2% of patients presented major bleeding. Finally, the 1-year results of TRILUMINATE-Pivotal, which were announced and simultaneously published at ACC 2023, included 350 patients, with 175 in the intervention and 175 in the medical therapy arm. The study showed that, regarding the primary composite endpoint of death from any cause or tricuspid-valve surgery, heart failure hospitalization and improvement of the Kansas City Cardiomyopathy Questionnaire (KCCQ) score of at least 15 points, this composite was favored in the TEER arm, with a win ratio of 1.48 (*p* = 0.02). Specifically, KCCQ score change was significantly higher in the TEER arm compared to the medical therapy arm; however, there was no significant difference in the incidence of death, tricuspid valve surgery or hospitalization for heart failure. In terms of procedural outcomes, 87.0% of the patients in the TEER arm and 4.8% of those in the control group had TR of no greater than moderate at 30 days (*p* < 0.001), while the intervention was found to be safe, as at 30 days follow-up, 98.3% of the patients who underwent the procedure were free of major adverse events [41].

#### 3.1.2. PASCAL

Fam et al. [42] were the first to study the effectiveness of the PASCAL system in TR. They included in their study 28 patients with severe TR, in whom a PASCAL device was implanted. The patients were followed up for 30 days. At the time of the follow-up, mortality rate was 7.1%. No major adverse cardiac and cerebrovascular events were observed. A total of 88% of patients were in NYHA functional class I or II, with TR grade ≤ 2+ in 85% at 30 days. In terms of safety, there were two single-leaflet device attachments, which were managed conservatively. The 6MWT distance was significantly improved. Another study by Kitamura et al. [43], in a similar size of patients, also found an improvement in TR severity, as 82% of patients had less than moderate TR after PASCAL implantation, as well as significant NYHA class and 6MWT distance improvement.

Kordali et al. [44], in the CLASP TR EFS, studied the PASCAL device in 34 patients with severe TR. A total of 97% of the patients had severe or greater TR, and 79% were NYHA III/IV at baseline. A total of 29 patients (85%) received PASCAL implants, and at 30 days follow-up, 85% of them achieved at least 1 TR grade reduction, with 52% having moderate or less residual TR (*p* < 0.001). A total of 89% of the patients were NYHA class I/II after the procedure (*p* < 0.001), while the mean 6MWT distance was significantly improved and the mean (KCCQ) score was improved by 15 points (*p* < 0.001). The rate of major adverse events was 5.9%. None of the patients experienced cardiovascular mortality, stroke, myocardial infarction, renal complication, or reintervention.

Recently, preliminary results from ongoing trials were also released for studies currently assessing the use of PASCAL in TR. Specifically, in EuroPCR 2022, the results of the 30 days of the TriCLASP study were reported [45], while the 6 months results were presented at PCR London Valves 2022 [46]. The study involved 74 individuals with severe symptomatic TR that received a PASCAL device and were followed up for 30 days and 6 months. A total of 72 patients finally underwent the procedure and 97% successfully received the device. At 30 days, 88% of patients had at least 1 TR grade reduction and 90% had moderate or lower TR. The composite major adverse event rate was 3.0%. At six months follow-up, 88% of patients had moderate or less TR, while 83% had at least 1 TR grade reduction. Furthermore, 61% of patients were NYHA I/II, while significant improvements were noted in both KCCQ score and 6MWT distance. The major adverse event rate at six months was 4.0%, while all-cause mortality was 5.1%. Moreover, Davidson presented the results of 30 days of the CLASP II TR trial from the roll in cohort [47] in TCT 2022. The trial included 73 patients followed up for 30 days. Data were available at the time of the follow-up for 68 patients. At 30 days, 83.0% of patients improved by 1 or more TR grade, 62.3% by 2 or more grades, and 73.6% had a TR class lower or equal to moderate TR. Statistically significant improvement was also noted in the NYHA class and the KCCQ score. In terms of safety, the composite major adverse event rate was 8.7%, with no mortality or heart failure hospitalizations reported. Finally, results of 1 year of the CLASP TR study [48] were also presented at EuroPCR 2022. The study originally enrolled 65 patients, however, 1-year follow-up data were available for 46. At the time of the follow-up, all patients improved by at least 1 and 75% by at least 2 TR grades, while 86% had moderate or lower TR. Significant improvement in the patients’ functional status were sustained at 1 year, as depicted by NYHA class and KCCQ score. The composite of major adverse events rate at 1 year was 16.9%, while all-cause mortality rate was 10.8%. With the use of the device, the annual rate of heart failure hospitalizations was reduced by 56.4%.

### 3.2. Annuloplasty

Transcatheter annuloplasty aims to improve TR taking into advantage the same mechanism of surgical annuloplasty, considering the pathophysiology of secondary TR; specifically, the annular dilation caused by the increased left heart pressures. Therefore, by reducing the annular diameter, this procedure aims to decrease the pathologic dilation and subsequently the regurgitation through the valve, providing the same clinical benefit of surgical annuloplasty via a transcatheter procedure. Several devices were tested (Table 2), with either suture-based or ring/non sutured devices, with Cardioband being the only device approved for use at the moment (CE Mark 2018). 

#### Cardioband

Nickenig et al. [49] were the first to study the use of Cardioband in the TRI-REPAIR trial. They enrolled 30 individuals with moderate to severe symptomatic TR unsuitable for surgical intervention, and a Cardioband was implanted in them. In all patients, the device was successfully implanted. Both at 30 days and 6 months, there was a sustained, significant reduction in the annulus diameter from baseline. This result was also significant at the 2 years follow-up [50]. Moreover, there was a reduction in the severity of TR, with 76% at 30 days, 73% at 6 months, and 72% at 2 years having less than moderate TR. A total of 88% of patients were NYHA class I/II at the 2 years follow-up. Finally, MWT and KCCQ score were both improved throughout the 2 years follow-up period.

In the TriBAND study [51], the researchers followed up 61 patients with severe functional TR who underwent a Cardioband implantation. The device was successfully implanted in 58 patients. Follow-up echocardiographic results were available for 54 and 42 patients at discharge and at 30 days follow-up, respectively. There was a 19% reduction in the annular diameter at discharge and 20% at 30 days. At least 1 TR class reduction was reported in 78% of patients at discharge and 85% of patients at 30 days, while 2 TR class reduction was achieved in 59% of patients at discharge and 30 days. Furthermore, 74% of patients were NYHA class I/II (*p* < 0.001). In terms of safety, all-cause mortality was 1.6%, while the rate of the composite of major adverse events was 19.7%. In PCR London Valves 2022, V. Rudolph presented the one-year results of the TriBAND study [52]. The tricuspid annulus diameter was reduced by 22% in one year, while 86% of patients had at least 1 TR grade reduction and 67% had at least a 2 TR grade reduction. A total of 61.1% of patients were NYHA class I/II at 1 year, while there was also a significant improvement in KCCQ score throughout the follow-up period. Cardiovascular mortality was 2.9%, while a major bleeding occurred in 11.5% of patients.

Following this, Davidson et al. [53], in the Cardioband TR Early Feasibility Study, studied 30 patients with a Cardioband and followed them up for 30 days. The device was successfully implanted in 28 patients. Annulus diameter was significantly decreased from baseline both at discharge and at follow-up. Furthermore, at 30 days, 85% of patients had a reduction of at least 1 TR class and 56% of at least 2 TR classes, while 44% had moderate or less TR (*p* < 0.001). Finally, 75% of patients were NYHA class I/II, while the KCCQ score at the follow-up was significantly improved. 

Finally, the 1-year results of the Cardioband TR Early Feasibility Study [54], including 37 patients, show a sustained reduction in TR as well as a sustained improvement in NYHA class and KCCQ score. In terms of safety, the 1-year cardiovascular-related mortality was 8.1%, while 5.4% of patients required reintervention, and in 35.1%, a major bleeding event was reported.

### 3.3. Tricuspid Valve Replacement

Transcatheter tricuspid valve replacement aims to percutaneously deliver a bioprosthetic tricuspid valve, mimicking an open-heart valve replacement surgery without the burden of a high-risk surgical procedure. This rapidly evolving treatment option seems to offer complete TR reduction and there is no concern regarding the most suitable leaflet anatomy, as opposed to the other previously discussed interventions. There are several devices currently being tested (Table 3), which will be discussed below.

#### 3.3.1. GATE

The first valve system used for transcatheter tricuspid valve replacement was the Gate system. Navia et al. [55] were the first to implant the Gate valve in two individuals with severe TR, not candidates for surgical management. The procedure was successful in terms of device implantation and TR reduction, with only mild paravalvular leaks being noted. Following this study, Hahn et al. [56] also showed a significant reduction in TR and in NYHA class. Finally, in the larger up-to-date study of the Gate valve [57], initially including 30 patients, the procedure was successful in 26 patients, while 2 patients (5%) were converted to open heart surgery. Out of the patients that successfully received the valve, all had a reduction in TR by at least 1 grade and 75% by at least 2 grades. At the time of the follow-up (mean follow-up time was 127 ± 82 days), 62% of patients were NYHA class I/II and four patients died. It is noteworthy that the in-hospital mortality rate was increased (10%); however, there were no late adverse outcomes related to the device at the time of the follow-up.

#### 3.3.2. Lux-Valve

Another device currently being assessed is the Lux-Valve. Lu et al. [58] reported the first use of this valve in humans in an observational study including 12 patients with severe TR. Procedural success was 100%. At 30 days follow-up, 90.9% of patients had no to mild residual TR, while 54.5% of patients were NYHA class II. There was one recorded death 18 days post procedurally due to vasospastic myocardial infarction, and one surgical reintervention for bleeding. Sun et al. [59], in a subsequent study, also reported great procedural success as well as echocardiographic and functional improvement at 1 year, with the exception of one mortality event due to heart failure worsening. 

Following this, T. Modine presented the one-year results of their multicenter study studying the Lux-Valve [60]. The study enrolled 31 patients and had a follow-up period up to one year. At the time of the follow-up, 92.9% of patients had mild or no TR and 100% had a reduction of at least 2 TR grades, while 82.8% were NYHA class I/II. The reported all-cause mortality at 1 year was 3.23%.

Lastly, the Lux-Valve Plus, a new system offering the same type of valve with a new delivery system permitting the transjugular approach, was recently tested in a first-in-man study [65]. The trial included 10 patients with a follow-up at 30 days. At the time of the follow-up, all patients had no or trivial TR (n = 90% and n = 10%, respectively) and a significant improvement in NYHA class (*p* < 0.05), while in terms of safety, there was no procedure-related, cardiovascular, cerebrovascular, or bleeding adverse events reported and the mortality at 30 days was 0%.

#### 3.3.3. Evoque

Evoque is another type of valve used for tricuspid valve repair. The first in-human use was reported by Fam et al. in a single case report, which was followed by a first in-human study by the same team [61], including 25 patients. The patients were followed up for 30 days. The procedural success was 92%, and no intraprocedural adverse outcomes were noted. A total of 96% had a TR grade lower or equal to 2, while 76% of patients were NYHA class I/II at the time of the follow-up. The mortality rate was 0%, and in terms of adverse effects, 12% of patients had a major bleeding, and in 8%, a pacemaker was implanted. Webb et al. [62] presented the 1-year follow-up results of the first in-human study. At 1 year, 97% of patients had a TR grade lower or equal to 2, while 87% had a TR grade of 1 or lower. A total of 70% of patients were NYHA class I/II at 1 year and the mortality rate was 7%.

Moreover, in the recently published results of 30 days of the TRISCEND trial [63], 56 patients were enrolled and received an Evoque valve. At 30 days, 98% of patients had mild or no TR and 78.8% of patients were NYHA class I/II. Furthermore, the KCCQ score and 6MWT distance were significantly improved. The composite major adverse event rate was 26.8%, with 1 cardiovascular death, 2 device embolization, and 15 severe bleedings that occurred at the time of the follow-up. The one-year results of the TRISCEND, report at PCR London Valves 2022 [64], showed that at one year, 97.6% of patients still had mild or no TR, while 93% remained NYHA class I/II. The significant improvements in the KCCQ score and 6MWT distance were sustained. Finally, the composite major adverse event rate was 30.2%, with 10.7% having experienced a major bleeding event.

### 3.4. Heterotropic Caval Valve Implantation

Hetertotropic caval valve implantation aims to implant a valvular device in the venae cavae system, which will reduce the systematic blood flow regurgitation as a result of the TR. In order for the procedure to apply to the patient, there must be presence of caval flow reversal and an appropriate inferior vena cava (IVC) diameter. There are three devices available, divided in two categories: balloon expandable devices (Sapien) and self-expandable devices (TricValve and Tricento). TricValve consists of two self-expandable valves, one in each vena cava, while Tricento has one valve implanted in the IVC. There is currently a limited number of trials regarding these systems (Table 4).

In respect to Sapien, the first randomized trial (TRICAVAL) was stopped due to safety concerns regarding valve dislocation62, while another non-randomized trial (HOVER) evaluating Sapien is currently being underwent [70].

In regards to TricValve, two randomized trials (TRICUS and TRICUS EURO) investigated the effect of TricValve in severe TR patients. TRICUS [67] evaluated the use of TR in 6 out of 24 patients, and even when not mentioning specific results for the device, in all patients, there was heart failure symptom relief and significant reduction in IVC and right atrium pressures. A total of 50.2% of patients functionally improved to NYHA class I/II. The thirty-day mortality rate was 8% and in-hospital mortality was 16%. The TRICUS EURO study [68] enrolled 35 patients in whom a TricValve was implanted. Thirty-day procedural success was 94% and no periprocedural deaths were recorded. At the six months follow-up, both KCCQ score and NYHA class were significantly improved, with 79.6% of patients being NYHA class I/II. Moreover, the six-month mortality rate was 8.5%.

Finally, in regards to the Tricento system, most experience is documented in case reports [71,72]. However, in a multicenter registry study [69], Wild et al. studied 21 patients receiving a Tricento prosthesis for a year, with a median follow-up of 61 days. The procedural success was 100%. Significant decrease was noted at both right ventricular end diastolic pressures and the NYHA class, with 65% of patients being NYHA I/II at the follow-up. In three patients, asymptomatic fractures of the device are noted. Lastly, the survival rate at one year was 76%.

## 4. Clinical Implications 

The evidence available from the aforementioned clinical trials suggests a safe and clinically efficient profile for transcatheter tricuspid valve interventions for both repair and valve replacement techniques. More specifically, most tested devices so far showed a good safety profile, with high rates of implantation, acute postprocedural and periprocedural success, and low rates of device-related adverse events or mortality. In addition, most devices show a significant improvement in TR severity, with most patients having a reduction of at least 1 grade in echocardiographic evaluation of TR severity and a large percent of 2 grades, with valve implantation showing the best results in terms of residual TR [73]. Finally, considering that most trials are not randomized and do not have a large follow-up period, the need for a large randomized trial in the near future comparing transcatheter interventions with optimal medical therapy and surgery, as well as transcatheter valve repair and replacement, should be highlighted.

**Figure 2:**
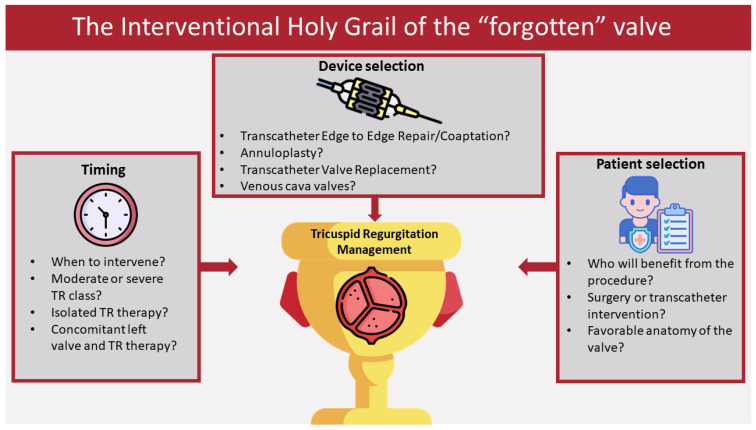
In the current era of tricuspid valve intervention, several parameters are of major significance for procedural success. The timing of the procedure should be carefully decided in regards to the course and severity of tricuspid regurgitation. The device selection has to be in accordance with each patient’s anatomy, as there is no “one size fits all” device. Finally, the patients who will benefit from transcatheter procedures should be carefully selected from a Heart Team, taking into consideration the surgical risk and the optimal intervention for each individual.

Given the large selection of devices and interventions currently being tested, it seems appropriate to adopt a personalized approach for each individual, with parameters such as patient selection, timing, and device selection being of great significance for the success of the operation (Figure 2). Patient selection highly depends on the anatomy of each individual’s tricuspid valve, the anatomy of which is highly complex [74]. Characteristics of the valve, such as leaflet number and morphology, are necessary information for the procedure, as the morphology of the valve may influence the success of the intervention [75]. It is noteworthy that there should be a careful procedural planning, as a result of the close proximity of the tricuspid annulus with vital cardiac structures (i.e., atrioventricular node), in order to avoid complications. It is equally important to properly identify those patients that could benefit from the procedure. In the most recent ESC/EACTS guidelines for valvular disease [11], transcatheter interventions for TR are recommended with an IIb class C recommendation only in symptomatic patients with severe secondary TR not suitable for surgery, in a center with expertise in transcatheter TR procedures, in whom improvement of quality of life or survival is to be expected. However, as more data and experience are gathered in the following years, these procedures may gain more indications for clinical use. In regards to timing, it is yet to be determined if transcatheter intervention in low grades of TR severity has any benefit in the clinical status of the patients, while it should also be discussed if the intervention should be combined with left heart valve intervention or if it should be an isolated procedure. Lastly, not all devices suit all patient anatomies; therefore, the device selection should be guided by the tricuspid morphology and each device’s characteristics. Even though there are not yet established indications and contraindications for certain procedures in regards to certain anatomic limitations of the tricuspid valve, Praz et al. [74] describe which devices better suit each anatomy, while they propose an algorithm for personalized device selection that could be useful in clinical decision making. Additionally, the implementation of dedicated risk scores for isolated TR interventions, such as TRI-SCORE, could be helpful in everyday clinical decision making and could guide healthcare professionals in optimizing the patient selection for either surgery or transcatheter interventions [76].

Finally, after the procedure, the patients should be appropriately followed up for evaluation of the procedural success. Follow-up should include an echocardiographic imaging of all patients, post procedurally and at 1 month, 3 months, 6 months, and 12 months after the intervention [74]. During follow-up visits, the appropriate pharmaceutical therapy should be prescribed in accordance with the patient’s symptoms and other related pathologies. Of particular interest is anticoagulation. There are not much clinical data or guideline-directed recommendations regarding the appropriate anticoagulation therapy yet, although some researchers suggest using the same anticoagulation regimen as for transcatheter mitral valve repair [74]. Regarding valve replacement, the same authors suggest a warfarin-/coumadin-based anticoagulation regimen, which could be combined with aspirin for at least one year. It should be, however, highlighted that there is uncertainty regarding the most appropriate anticoagulation regimen, as the bleeding risk is increased in these patients. Therefore, more experience and trials addressing this issue are required.

## 5. Future Directions

Currently, there are numerous trials underway, with several of them being randomized, aiming to further examine the safety and efficacy of transcatheter tricuspid valve interventions, as well as to compare them with optimal medical therapy. However, as clinical data continue to congregate, clinical experience on the approved devices should be gained by more heart teams. Therefore, educational opportunities or interventional workshops from experienced centers should be organized, while educational modules for the periprocedural imaging of the tricuspid valve using echocardiography should also be planned, as echocardiographic guidance is of major significance in interventional structural procedures. These strategies could further promote the use of transcatheter tricuspid intervention in more centers, eventually reaching more patients suitable for these procedures.

## 6. Conclusions

Transcatheter TR interventions offer a safe and efficient option for treating TR, providing promising results in clinical trials. Even though currently only selected centers have experience with this procedure, as more devices are approved by regulatory authorities, more interventions will begin to take place. Therefore, cardiologists should be aware of the patients indicated for percutaneous TR treatments, as well as the data supporting the devices they use, in order to safely implement these interventions into their practice.

## Figures and Tables

**Table 1:** Trials regarding transcatheter edge-to-edge repair (TEER) of the tricuspid valve.

Author	Device	Participants (Age)	Follow-Up Time	Outcomes			
				TR Reduction	NYHA Class	6MWT/KCCQ Score	Safety Endpoints
Nickenig et al. [33]	MitraClip	64 (76.6)	30 days	A total of 91% of patients had at least 1 TR grade reduction, and 13% of patients remained in severe/massive TR; however, there was a significant decrease compared to baseline (88%) (*p* = 0.01).	No patients were NYHA IV; however, 63% remained NYHA III.	A 6 min walking test was significantly improved after the procedure (16.1 m increase; *p* = 0.007).	The device was implanted successfully in 97% of patients. There were no intraprocedural deaths. In-hospital mortality rate was 5%.
Orban et al. [34]	MitraClip	50 (77)	6 months	A total of 90% of patients had a reduction of at least 1 TR grade and 77% had a TR grade lower than 2.	NYHA class was improved by at least 1 class in 79% of patients.	A 6MWT distance was found to be increased by 44% (84 m, *p* = 0.056) and KCCQ score was improved by 21 points (*p* < 0.0001).	The event free survival rate was 78%. There was a total of 28% of patients hospitalized for worsening heart failure during the follow-up period.
Braun et al. [35]	MitraClip	31 (77)	30 days	A total of 69% of patients had a TR grade lower or equal to 2.	A total of 69% of patients were NYHA class I/II (*p* < 0.001).	Not mentioned.	A total of 1 patient died due to insufficient TR reduction.
Mehr et al. (TriValve registry) [36]	TriClip	249 (77)	1 year	TR reduction was sustained in 84% of patients, and 72% of patients had a TR grade lower or equal to 2 at the time of the follow-up.	A total of 69% of patients were NYHA I/II at the follow-up (*p* < 0.001).	Not mentioned.	Implantations success rate was 96%. The combined endpoint of death or unplanned hospitalization for heart failure occurred in 31% of patients. One year mortality rate was 20.3%.
Lurz et al. (TRILUMINATE) [37]	TriClip	85 (77.8)	1 year	At 1 year, 87% of subjects had a sustained TR reduction of at least 1 TR severity grade, and 70% of subjects had moderate or less TR at the time of the follow-up.	A total of 83% of patients were NYHA I/II at 1 year.	A 6MWT distance increased from by 31 m at 1 year.	Hospitalization rate decreased from 1.30 to 0.78 events/patient–year.
Lurz et al. (bRIGHT study) [38]	TriClip	300 (78.5)	30 days	A total of 71% of patients had moderate or less TR (*p* < 0.0001).	A total of 78% of patients were NYHA class I/II (*p* < 0.0001).	There was a significant improvement in KCCQ score by 18 points (*p* < 0.0001).	Implantation success was 98%. Major adverse event rate was 1%.
Lurz et al. (bRIGHT study) [39]	TriClip	200 (78)	1 year	A total of 86% of patients had moderate or less TR at 1 year follow-up.	A total of 77% of patients were NYHA class I/II.	KCCQ score was significantly improved by 21 points (*p* < 0.0001).	The major adverse event rate was 11.5%. All-cause mortality was 11.0%. There was a 44% reduction in heart failure hospitalizations during the follow-up, compared to the year before the intervention.
Adams et al. (TRILUMINATE-Pivotal study) [40]	TriClip	97 (79)	30 days	A total of 74% of patients had less than moderate TR, and 67% of patients had a reduction in TR class of at least 2 grades.	A total of 76% of patients were NYHA I/II.	KCCQ score was significantly improved by 16.64 points.	The implantation success was 99%. Mortality rate was 1% 7.2% had a major bleeding during the follow-up.
Sorajja et al. (TRILUMINATE-Pivotal 1 year follow-up) [41]	TriClip	350, 175 in each arm (TriClip vs. medical therapy) (78)	30 days 1 year	TEER arm: 87.0% of the TR of no greater than moderate at 30 days. Medical therapy arm: 4.8% of the TR of no greater than moderate at 30 days.	TEER arm: 83.9% of patients were NYHA I/II at 1 year follow-up. Medical therapy arm: 59.5% of patients were NYHA I/II at 1 year follow-up.	TEER arm: KCCQ score was improved by a mean of 12.3 ± 1.8 points. Medical therapy arm: KCCQ score was improved by a mean of 0.6 ± 1.8 point.	A total of 98.3% of the patients who underwent the procedure were free from major adverse events at the 30 day follow-up.
Fam et al. [42]	PASCAL	28 (78)	30 days	A total of 85% of patients had a TR grade lower or equal to 2.	A total of 88% of patients were NYHA class I/II at the follow-up.	A 6MWT distance was increased by 95 m (*p* < 0.001).	Mortality rate was 7.1%.
Kitamura et al. [43]	PASCAL	30 (77)	1 year	TR reduction of at least 1 grade was sustained in 89% of patients at 1 year, and 82% and 86% of patients had moderate or less TR at the 30 day and 1 year, respectively.	A total of 90% of patients were NYHA class I/II.	A 6MWT distance was increased by72 m at 1 year (*p* < 0.01).	A 1 year survival rate was 93%. A total of 20% of patients required a rehospitalization due to acute heart failure during the 1 year follow-up period. Cardiovascular mortality rate was 6.7%.
Kodali et al. [44]	PASCAL	34 (76)	30 days	A total of 85% of patients had a TR severity reduction of at least 1 grade at 30 days, and 52% had moderate or less TR severity (*p* < 0.001).	A total of 89% of the patients followed up were NYHA class I/II (*p* < 0.001).	A 6MWT distance was improved by 71 m (*p* < 0.001) and KCCQ score improved by 15 points (*p* < 0.001).	The major adverse events rate was 5.9%.
Baldus et al. (TriClasp study) [45]	PASCAL	74(with 72 undergoing the procedure) (80)	30 days	A total of 88% of patients achieved at least 1 TR grade reduction, and 90% had moderate or lower TR severity.	A total of 56% of patients were NYHA class I/II.	A 6MWT distance was significantly improved by 38 m (*p* < 0.001) and KCCQ scire was significantly improved by 13 points (*p* < 0.001).	Successful implantation rate was 97%. All-cause mortality rate was 2.9%. Composite major adverse event rate was 3.0%. Cardiovascular mortality rate was 1.5%. Heart failure rehospitalization rate was 4.5%.
Schofer et al. (TriClasp study) [46]	Pascal	177 (80)	6 months	A total of 88% of patients had moderate or lower TR, and 83% of patients achieved equal or greater than 1 TR grade reduction at 6 months.	A total of 61% of patients were NYHA class I/II.	A 6MWT distance was improved by 32 m (*p* = 0.01) and KCCQ score was improved by 9 points (*p* < 0.001).	Implantation success was 99%. The composite major adverse events rate was 4%. All-cause mortality rate was 5.1%. Cardiovascular mortality rate was 2.3%. The rate of heart failure hospitalization was 7.9% at 6 months.
Davidson et al. (CLASP II TR roll in) [47]	PASCAL	73 (79.8)	30 days	A total of 83.0% of patients improved by 1 or more TR grade, 62.3% improved by 2 or more grades, and 73.6% of patients had moderate or less TR severity.	A total of 86% of patients were NYHA class I/II.	KCCQ score was significantly improved by 17.9 points (*p* < 0.001).	The rate of successful implantation was 84.4%. The composite major adverse events rate was 8.7%. Cardiovascular mortality rate was 0%. All-cause mortality and heart failure hospitalization rate were both 0%.
Hahn et al. (CLASP TR) [48]	PASCAL	65 (46 had 1 year follow-up) (77)	1 year	A total of 100% of patients improved by at least 1 TR grade, while 75% improved by at least 2 TR grades, and 86% of patients had moderate or less TR severity at 1 year.	A total of 92% of patients were NYHA class I/II.	A 6MWT distance was significantly increased by 94 m (*p* < 0.014). KCCQ score was significantly improved by 18 points (*p* < 0.001).	The implantation success rate was 91%. The composite major adverse events rate was 16.9%. All-cause mortality rate was 10.8%. Cardiovascular mortality rate was 7.7%. Heart failure rehospitalization rate was 18.5%.

**Table 2:** Trial regarding transcatheter tricuspid valve annuloplasty techniques.

Author	Device	Participants (Age)	Follow-Up Time	Outcomes			
				Annulus Reduction TR Reduction	NYHA Class	6 MWT Distance/KCCQ Score	Safety Endpoints
Nickenig et al. (TRI-REPAIR study) [49]	Cardioband	30 (75)	30 days 6 months	Significant reduction in the annulus at 30 days (37.8 ± 3.3 mm; *p* = 0.0004) at 6 months (37.8 ± 3.4 mm; and *p* = 0.0014) from baseline (41.9 ± 4.6 mm). A total of 76% at 30 days and 73% at 6 months had less than moderate TR.	A total of 82% at 30 days and 88% at 6 months were NYHA class I/II.	KCCW score was significantly improved by 12 at 30 days and 24 points at 6 months. The 6MWT distance was significantly improved by 31 m at 30 days and 60 m at 6 months.	The implantation was 100% successful. All-cause morality at 6 months was 10%.
Nickenig et al. (TRI-REPAIR study) [50]	Cardioband	30 (75)	2 years	Significant reduction in the annulus sustained at 2 years follow-up (35.2 ± 4.6 mm, *p* < 0.001) from baseline (41.9 ± 4.6 mm). A total of 72% at 2 years had less than moderate TR.	A total of 82% were NYHA class I/II.	KCCW score was significantly improved by 18 points at 2 years. The 6MWT distance was significantly improved by 63 m at 2 years.	The two-year survival rate was 73%. The two-year freedom from heart failure hospitalization rate was 55%. At two years, there were eight deaths. Two patients underwent device-related secondary procedures.
Nickenig et al. (TriBand study) [51]	Cardioband	61 (78.6)	30 days	A total of 69% (*p* < 0.001) of patients achieved less than moderate TR, and 85% of patients had at least 1 TR grade reduction. Annular diameter was reduced by 20%.	A total of 74% were in NYHA class I-II (*p* < 0.001).	KCCQ score was significantly improved by 17 points (*p* < 0.001).	Device success rate was 96.7%. All-cause mortality rate was 1.6%. The composite major adverse event rate was 19.7%. Cardiovascular mortality was 0%.
Rudolph et al. (TriBand study) [52]	Cardioband	139 (79) (62 available at follow-up)	1 year	The annulus diameter was decreased by 22%, 86% of patients achieved greater or equal to 1 TR grade reduction, 67% had greater or equal to a 2 TR grade reduction, and 77.5% of patients had moderate or less TR severity (*p* < 0.001).	A total of 61.1% of patients were NYHA class I/II (*p* < 0.001).	KCCQ score was significantly improved by greater than 20 points in 32% and 10–19 points in 18% (*p* < 0.001).	Device success was 93%. The composite major adverse event rate was 20.9%. Cardiovascular mortality was 2.9%. Survival rate was 92%. Freedom from HF hospitalization was 84%.
Davidson et al. (Cardioband TR EFS) [53]	Cardioband	30 (77)	30 days	The annulus diameter was decreased by 5.7 mm (*p* < 0.001). A total of 85% of patients had at least 1 TR grade reduction, 56% of patients had at least 2 TR grade reduction, and 44% of patients had moderate or less TR severity.	A total of 75% of patients were NYHA class I/II (*p* < 0.001).	KCCQ score improved by 16 points (*p* < 0.001). The 6MWT distance was not significantly different from baseline.	The device was successfully implanted in 93.3% of patients. Cardiovascular and estimated all-cause mortality were 0%.
Gray et al. (Cardioband TR EFS) [54]	Cardioband	37 (78)	1 year	Annular diameter was significantly decreased by 10.5 mm (*p* < 0.0001), 73.0% of patients had less than moderate TR severity(*p* < 0.0001), and 73.1% of patients had at least a reduction of 2 TR grades.	A total of 92.3% of patients were NYHA class I/II.	KCCQ score was significantly increased by 19.0 points (*p<* 0.0001).	Cardiovascular mortality rate was 8.1%. One year survival rate was 85.9%. One year freedom from heart failure rehospitalization rate was 88.7%.

**Table 3:** Trial regarding transcatheter tricuspid valve replacement.

Author	Device	Participants (Age)	Follow-Up Time	Outcomes			
				TR Reduction	NYHA Class	6MWT Distance/KCCQ Score	Safety Endpoints
Navia et al. [55]	GATE	2 (71)	3–6 months	Both patients had mild paravalvular leak.	Not mentioned.	Not mentioned.	Not mentioned.
Hahn et al. [56]	GATE	5 (84,4) (follow-up for 4/5 patients)	3–6 months	In three patients, trace to mild TR was noticed after the procedure, while in one patient, it was mild to moderate. Mild paravalvular leaks did not change in the course of the follow-up.	Not mentioned.	Not mentioned.	Implantation success was 100%. There was one death post operatively (day 28).
Hahn et al. [57]	GATE	30 (75)	30 days	A total of 95% of patients had at least 1 TR grade reduction, and 81% of patients had at a least 2 TR grade reduction in TR severity at 30 days. A total of 74% of patients had mild or less TR at follow-up (*p* < 0.001).	A total of 77% were NUHA class I/II (*p* < 0.005).	Not mentioned.	Implantation success was 87%. A total of 5% of procedures turned into open heart surgery due to device malposition. In-hospital mortality rate was 10%.
Lu et al. [58]	Lux Valve	12 (69)	30 days	A total of 90.9% of patients had no to mild TR at follow-up.	A total of 54.5% were NYHA class II; (*p* < 0.05).	The 6MWT distance was improved by 99.5 m.	Procedural success was 100%. There was a total of one cardiovascular-related death during the follow-up period.
Sun et al. [59]	Lux Valve	6 (56)	12 months	There was moderate paravalvular regurgitation in one patient, mild paravalvular regurgitation in three patients, and no paravalvular regurgitation in two patients. There was no change in paravalvular leak severity at 1 year.	All alive patients (5/6) were NYHA class I/II at 1 year.	Not mentioned.	Implantation success 100%. There was 0% intraprocedural mortality at 30 days follow-up, And one patient died at 3 months due to right ventricular failure.
Modine et al. [60]	Lux Valve	31 (67.8)	1 year	All patients had no TR at 30 days follow-up, and 92.9% of patients had mild or no TR at 1 year follow-up.	A total of 82.8% of patients were NYHA class I/II.	Not mentioned.	All-cause mortality rate was 3.23% at follow-up. Survival rate was 96.8% at 1 year.
Fam et al. [61]	Evoque	25 (76)	30 days	TR grade was less or equal to 2 in 96% of patients at 30 days follow-up.	A total of 76% of patients were NYHA class I/II.	Not mentioned.	Implantation success rate was 92%. There was a 0% mortality rate at 30 days follow-up. There was a 0% intraprocedural mortality or conversion to open surgery.
Webb et al. [62]	Evoque	27 (77)	1 year	A total of 96% of patients had a TR grade lower or equal to 2, and 87% of patients had a TR grade lower or equal to 1.	A total of 70% of patients were NYHA class I/II.	Not mentioned.	Mortality rate was 7%. Two patients required hospitalization for heart failure.
Kodali et al. (TRISCEND study) [63]	Evoque	56 (79.3)	30 days	A total of 98.1% of patients had mild or no/trace TR severity (*p* < 0.001).	A total of 78.8% of patients were NYHA class I/II (*p* < 0.001).	The 6MWT distance was increased by 48.2 m (0.001). KCCQ score was improved by 19.0 points (*p* < 0.001).	The composite major adverse events rate was 26.8%.
Windecker et al. (TRISCEND study) [64]	Evoque	176 (78.7)		A total of 97.6% of patients had lower than mild TR severity at 1 year, and 69% of patients had non/trace TR.	A total of 93% of patients were NYHA class I/II.	The 6MWT distance was increased by 25.7 m (*p* < 0.001). KCCQ score was improved by 25.7 points (*p* < 0.001) 55%of patients had more or equal to a 20-point improvement in the KCCQ score, and 22% of patients had 10–19 points of improvement in KCCQ.	The composite major adverse events rate at 1 year was 30.2%. Survival rate was 90%. Freedom from heart failure hospitalization rate was 88%.

**Table 4:** Trials regarding heterotopic caval valve implantation.

Author	Device	Participants (Age)	Follow-Up Time	Outcomes		
				NYHA Class	6MWT Distance/KCCQ Score	Safety Endpoints
Dreger et al. [66]	Sapien	28 (14 intervention vs. 14 medical therapy) (77)	1 year	The trial was stopped due to safety concerns (valve dislocations) at 3 months. However, a significant (*p* = 0.025) improvement in NYHA class was reported.		
Lauten et al. (TRICUS) [67]	TricValve and others	25 (73.9) (TtricValve = 6)	1 year	A total of 52.7% were NYHA class I/II.	Not mentioned.	The 30-day mortality was 8%. In-hospital mortality was 16%.
Estévez-Loureiro (TRICUS EURO study) [68]	TricValve	35 (76)	30 days and 6 months	A total of 79.4% of patients were NYHA class I/II at 6 months (*p* = 0.0006).	KCCQ score was improved by 17.7 points at 6 months (*p* = 0.004).	All-cause mortality rate was 8.5%. Heart failure hospitalization rate was 20%.
Wild et al. [69]	Tricento	21 (76)	1 year	A total of 65% of patients were NYHA class I/II.	Not mentioned.	Technical success was 100%. In-hospital mortality was 0%. The 1-year survival rate was 76%. Heart failure rehospitalization rate was 19% at 1 year.

## Data Availability

Review data available under request from the authors.
